# Supplementary material for: In-hospital outcomes and 30-day readmission rates among ischemic and hemorrhagic stroke patients with delirium
Source: PLoS One. 2019 Nov 14;14(11):e0225204. doi: 10.1371/journal.pone.0225204 (PMC6855446; doi:10.1371/journal.pone.0225204)
Supplement: S1 Table — (DOCX) [file pone.0225204.s001.docx]

**S1 Table. ICD-9 codes for delirium.**

|  | **ICD-9 Codes** | **Comparison** | **Sensitivity** | **Specificity** | **PPV** | **NPV** |
| --- | --- | --- | --- | --- | --- | --- |
| **Kim 2017 [1]** | 290.11, 290.12, 290.13, 290.2, 290.3, 290.41, 290.42, 290.43, 290.8, 290.9, 291, 292, 292.11, 292.12, 292.2, 292.81, 292.82, 293, 293.1, 293.81, 293.82, 293.83, 293.84, 293.89, 293.9, 348.3, 348.31, 348.39, 349.82, 780.02, 780.09, 780.97 | CAM | 20 | 99 | 91 | 66 |
| **Bui 2016 [2]** | 290.11, 290.12, 290.13, 290.2, 290.3, 290.41, 290.42, 290.43, 290.8, 290.9, 291, 292, 292.1, 292.2, 292.81, 292.82, 293, 293.1, 293.8, 293.9, 348.3, 348.31, 348.39, 349.82, 780.02, 780.09 | CAM-ICU | 36 | 95 | 83 | 69 |
| **Hope 2014 [3]** | 290.3, 291, 291.1, 292, 292.81, 293, 293.1, 780.09 | Expert Consensus | 28 | NR | NR | NR |
| **Lin 2010 [4]** | 290.11, 290.3, 290.41, 292.81, 293, 293.1 | NR | NR | NR | NR | NR |
| **Lin 2010 (dementia) [4]** | 290.11, 290.3, 290.41 | NR | NR | NR | NR | NR |
| **Lin 2010 (drug) [4]** | 292.81 | NR | NR | NR | NR | NR |
| **Lin 2010 (NDND) [4]** | 293, 293.1 | NR | NR | NR | NR | NR |
| **Inouye 2005 [5]** | 290.11, 290.3, 290.41, 291, 292.81, 293, 293.1, 780.09 | CAM | 3 | 99 | NR | 88 |
| **Johnson 1992 [6]** | 290.11, 290.3, 290.41, 291, 292, 292.1, 292.12, 292.2, 292.81, 292.9, 293, 293.1, 293.9, 780 | DSM III | 9 | NR | NR | NR |

PPV, positive predictive value; NPV, negative predictive value; NR, Not Reported

**S1 Table References**

1. Kim DH, Lee J, Kim CA, Huybrechts KF, Bateman BT, et al. (2017) Evaluation of algorithms to identify delirium in administrative claims and drug utilization database. Pharmacoepidemiol Drug Saf 26: 945-953.

2. Hendrickx JF, De Wolf A, Skinner S (2016) Journal of Clinical Monitoring and Computing 2015 end of year summary: anesthesia. J Clin Monit Comput 30: 1-5.

3. Hope C, Estrada N, Weir C, Teng CC, Damal K, et al. (2014) Documentation of delirium in the VA electronic health record. BMC Res Notes 7: 208.

4. Lin RY, Heacock LC, Fogel JF (2010) Drug-induced, dementia-associated and non-dementia, non-drug delirium hospitalizations in the United States, 1998-2005: an analysis of the national inpatient sample. Drugs Aging 27: 51-61.

5. Inouye SK, Leo-Summers L, Zhang Y, Bogardus ST, Jr., Leslie DL, et al. (2005) A chart-based method for identification of delirium: validation compared with interviewer ratings using the confusion assessment method. J Am Geriatr Soc 53: 312-318.

6. Johnson JC, Kerse NM, Gottlieb G, Wanich C, Sullivan E, et al. (1992) Prospective versus retrospective methods of identifying patients with delirium. J Am Geriatr Soc 40: 316-319.
