# Supplementary material for: In-hospital outcomes and 30-day readmission rates among ischemic and hemorrhagic stroke patients with delirium
Source: PLoS One. 2019 Nov 14;14(11):e0225204. doi: 10.1371/journal.pone.0225204 (PMC6855446; doi:10.1371/journal.pone.0225204)
Supplement: S2 Table — (DOCX) [file pone.0225204.s002.docx]

**S2 Table. Descriptive and univariate analysis for all stroke types by delirium status (January 2010 to September 2015).**

|  | **Total**  **(n = 3,107,437)** | **No Delirium**  **(n = 2,875,938)** | **Delirium**  **(n = 231,500)** | **OR (95% CI)** |
| --- | --- | --- | --- | --- |
| **Demographic Characteristics** | | | | |
| Age – mean (SE) | 70.12 (0.07) | 69.85 (0.07) | 73.41 (0.09) | 1.02 (1.02-1.02) |
| Age Category – years (%) | | | | |
| 18 – 64 | 34.28 | 34.98 | 25.58 | REF |
| 65 - 80 | 36.39 | 36.43 | 35.89 | 1.35 (1.32-1.38) |
| 81 - 90 | 29.32 | 28.58 | 38.53 | 1.84 (1.80-1.89) |
| Female (%) | 51.71 | 51.53 | 53.98 | 1.10 (1.09-1.12) |
| Insurance (%) | | | | |
| Medicare | 65.72 | 65.03 | 74.29 | REF |
| Medicaid | 8.04 | 8.08 | 7.51 | 0.81 (0.79-0.84) |
| Private | 18.36 | 18.84 | 12.47 | 0.58 (0.56-0.60) |
| Other | 7.88 | 8.06 | 5.72 | 0.62 (0.60-0.64) |
| Patient Residential County (%) | | | | |
| “Central” Large Metro | 24.99 | 24.98 | 25.09 | REF |
| “Fringe” Large Metro | 25.10 | 25.09 | 25.15 | 1.00 (0.96-1.04) |
| Other (Non-Large Metro) | 49.91 | 49.93 | 49.76 | 0.99 (0.96-1.03) |
| Median Household Income for Patient ZIP Code (Quartile) | | | | |
| $1-37,999 | 31.37 | 31.33 | 31.93 | REF |
| $38,000-47,999 | 25.65 | 25.66 | 25.59 | 0.98 (0.95-1) |
| $48,000-63,999 | 23.30 | 23.32 | 23.03 | 0.97 (0.94-1) |
| ≥$64,000 | 19.67 | 19.69 | 19.45 | 0.97 (0.94-1) |
| **Comorbidities (%)** | | | | |
| Atrial Fibrillation | 23.47 | 23.02 | 29.03 | 1.37 (1.34-1.39) |
| Coagulopathy | 4.03 | 3.84 | 6.42 | 1.72 (1.66-1.77) |
| Hypertension | 82.24 | 82.24 | 82.23 | 1.00 (0.98-1.02) |
| Other neurologic disorder | 2.21 | 1.96 | 5.33 | 2.81 (2.7-2.93) |
| Alcohol | 4.75 | 4.51 | 7.64 | 1.75 (1.70-1.80) |
| Anemia | 13.35 | 12.85 | 19.58 | 1.65 (1.62-1.69) |
| Chronic Blood loss | 0.41 | 0.40 | 0.62 | 1.56 (1.42-1.72) |
| Congestive Heart Failure | 13.68 | 13.27 | 18.79 | 1.51 (1.48-1.54) |
| Chronic Pulmonary Disease | 15.04 | 14.89 | 16.92 | 1.16 (1.14-1.19) |
| Depression | 11.22 | 11.07 | 13.06 | 1.21 (1.18-1.24) |
| Diabetes Mellitus | 28.78 | 28.83 | 28.12 | 0.97 (0.95-0.98) |
| Diabetes with complications | 6.48 | 6.38 | 7.79 | 1.24 (1.21-1.28) |
| Drug Abuse | 2.90 | 2.78 | 4.40 | 1.61 (1.55-1.67) |
| Liver Disease | 1.46 | 1.39 | 2.22 | 1.61 (1.53-1.69) |
| Fluid and electrolyte disorders | 24.74 | 23.30 | 42.52 | 2.43 (2.39-2.48) |
| Obesity | 10.36 | 10.47 | 9.05 | 0.85 (0.83-0.88) |
| Peripheral Vascular Disease | 9.38 | 9.31 | 10.22 | 1.11 (1.08-1.14) |
| Psychoses | 3.76 | 3.52 | 6.71 | 1.97 (1.91-2.04) |
| Disorder of pulmonary circulation | 3.50 | 3.40 | 4.75 | 1.42 (1.37-1.47) |
| Renal Failure | 14.27 | 13.88 | 19.13 | 1.47 (1.44-1.50) |
| Solid Tumor without Metastasis | 1.78 | 1.76 | 2.12 | 1.21 (1.15-1.27) |
| Ulcer | 0.03 | 0.03 | 0.03 | 1.03 (0.72-1.48) |
| Valvular disorders | 9.29 | 9.25 | 9.88 | 1.08 (1.05-1.11) |
| Number of chronic conditions – mean (SE) | 7.32 (0.02) | 7.22 (0.02) | 8.55 (0.03) | 1.10 (1.10-1.10) |
| Charlson Comorbidity Index – mean (SE) | 3.35 (0.01) | 3.31 (0.01) | 3.77 (0.01) | 1.09 (1.08-1.09) |
| Charlson Comorbidity Index (%) | | | | |
| 1 | 23.80 | 24.31 | 17.47 | REF |
| ≥ 2 | 76.20 | 75.69 | 82.53 | 1.52 (1.49-1.55) |
| All Patient Refined DRG mortality (Likelihood of dying) (%) | | | | |
| Minor | 26.11 | 27.69 | 6.01 | REF |
| Moderate | 42.31 | 43.30 | 29.78 | 3.17 (3.05-3.29) |
| Major | 19.15 | 17.66 | 38.15 | 9.95 (9.53-10.38) |
| Extreme | 12.42 | 11.35 | 26.04 | 10.56 (10.08-11.07) |
| **Disease Severity and Treatment (%)** | | | | |
| All Patient Refined DRG severity of illness (Loss of function) (%) | | | | |
| Minor | 10.82 | 11.50 | 2.06 | REF |
| Moderate | 44.51 | 46.14 | 23.73 | 2.87 (2.69-3.05) |
| Major | 32.75 | 31.71 | 45.98 | 8.08 (7.60-8.60) |
| Extreme | 11.91 | 10.64 | 28.20 | 14.78 (13.84-15.78) |
| Stroke Type (%) | | | | |
| Ischemic Stroke | 84.27 | 84.70 | 78.97 | REF |
| Intracerebral Hemorrhage | 11.39 | 11.08 | 15.31 | 1.48 (1.45-1.52) |
| Sub Arachnoid Hemorrhage | 4.33 | 4.22 | 5.71 | 1.45 (1.39-1.52) |
| Intravenous tPA | 5.96 | 6.07 | 4.63 | 0.75 (0.72-0.78) |
| Intra-Arterial Therapy | 1.06 | 1.06 | 1.10 | 1.04 (0.96-1.13) |
| Gastric Tube | 4.94 | 4.56 | 9.72 | 2.25 (2.18-2.33) |
| Extra Ventricular Drain | 1.55 | 1.42 | 3.18 | 2.27 (2.14-2.41) |
| Hemicraniectomy / Hemicraniotomy | 0.48 | 0.46 | 0.80 | 1.75 (1.60-1.92) |
| Ventilator Support | 0.02 | 0.02 | 0.05 | 2.93 (2.07-4.15) |
| Tracheostomy | 1.36 | 1.24 | 2.90 | 2.38 (2.25-2.52) |
