# Supplementary material for: In-hospital outcomes and 30-day readmission rates among ischemic and hemorrhagic stroke patients with delirium
Source: PLoS One. 2019 Nov 14;14(11):e0225204. doi: 10.1371/journal.pone.0225204 (PMC6855446; doi:10.1371/journal.pone.0225204)
Supplement: S3 Table — (DOCX) [file pone.0225204.s003.docx]

**S3 Table**: **In-hospital outcomes for stroke patient discharges with and without delirium by stroke-type.**

(a) Ischemic Stroke

|  | **Total**  **(n = 2,618,762)** | **No Delirium**  **(n = 2,435,942)** | **Delirium**  **(n = 182,820)** | **aOR / aRR**  **(95% CI)*** |
| --- | --- | --- | --- | --- |
| Died, % (95% CI) | 5.10  (5.02 – 5.18) | 4.71  (4.63 – 4.79) | 10.29  (9.99 – 10.60) | 1.80  (1.74 – 1.87) |
| Length of Stay, mean (SE) | 6.92 (0.03) | 6.67 (0.03) | 10.26 (0.07) | 1.22  (1.21 – 1.23) |
| Discharge Disposition, % (95% CI) | | | | |
| Home incl. Home with Home Health | 58.32  (58.00 – 58.64) | 59.99  (59.66 – 60.31) | 36.08  (35.57 – 36.59) | Reference |
| Transfer (Hosp /SNF/ICF/Other) | 35.68  (35.36 – 36.00) | 34.40  (34.07 – 34.72) | 52.78  (52.25 – 53.30) | 1.83  (1.78 - 1.87) |
| Died | 5.10  (5.02 – 5.18) | 4.71  (4.63 – 4.79) | 10.29  (9.99 – 10.60) | 2.46  (2.36 - 2.56) |
| Other | 0.90  (0.87 – 0.93) | 0.90  (0.87 – 0.93) | 0.85  (0.77 – 0.94) | 1.59  (1.44 - 1.74) |

aOR, adjusted odds ratio; aRR, adjusted risk ratio; Hosp, acute care hospitalization; SNF, skill nursing factility; ICF, intermediate care facility.

* adjusted risk ratio and 95% CI reported for length of stay

(b) Intracerebral Hemorrhage

|  | **Total**  **(n = 354,037)** | **No Delirium**  **(n = 318,583)** | **Delirium**  **(n = 35,454)** | **aOR / aRR**  **(95% CI)*** |
| --- | --- | --- | --- | --- |
| Died, %(95% CI) | 25.92  (25.60 – 26.24) | 26.49  (26.15 – 26.83) | 20.84  (20.01 – 21.70) | 0.83  (0.78 - 0.88) |
| Length of Stay, mean(SE) | 10.01 (0.07) | 9.43 (0.07) | 15.23 (0.24) | 1.30  (1.27 - 1.33) |
| Discharge Disposition, % (95% CI) | | | | |
| Home incl. Home with Home Health | 35.03  (34.58 – 35.47) | 35.91  (35.45 – 36.37) | 27.09  (26.17 – 28.02) | Reference |
| Transfer (Hosp /SNF/ICF/Other) | 38.43  (37.97 – 38.90) | 36.99  (36.51 – 37.48) | 51.35  (50.31 – 52.39) | 1.54  (1.46 - 1.63) |
| Died | 25.92  (25.60 – 26.24) | 26.49  (26.15 – 26.83) | 20.84  (20.01 – 21.70) | 1.05  (0.99 - 1.13) |
| Other | 0.62  (0.56 – 0.69) | 0.61  (0.54 – 0.67) | 0.72  (0.59 – 0.89) | 1.44  (1.17 - 1.79) |

aOR, adjusted odds ratio; aRR, adjusted risk ratio; Hosp, acute care hospitalization; SNF, skill nursing factility; ICF, intermediate care facility.

* adjusted risk ratio and 95% CI reported for length of stay

(c) Subarachnoid Hemorrhage

|  | **Total**  **(n = 134,630)** | **No Delirium**  **(n = 121,413)** | **Delirium**  **(n = 13,226)** | **aOR / aRR**  **(95% CI)*** |
| --- | --- | --- | --- | --- |
| Died, %(95% CI) | 20.36  (19.83 – 20.90) | 20.75  (20.20 – 21.32) | 16.77  (15.62 – 17.99) | 0.75  (0.69 - 0.83) |
| Length of Stay, mean(SE) | 13.87 (0.12) | 13.13 (0.12) | 20.70 (0.32) | 1.27  (1.24 – 1.31) |
| Discharge Disposition, % (95% CI) | | | | |
| Home incl. Home with Home Health | 53.23  (52.46 – 53.99) | 54.55  (53.81 – 55.29) | 41.12  (39.33 – 42.94) | Reference |
| Transfer (Hosp /SNF/ICF/Other) | 25.87  (25.14 – 26.61) | 24.15  (23.43 – 24.88) | 41.68  (40.09 – 43.29) | 1.74  (1.58 - 1.9) |
| Died | 20.36  (19.83 – 20.90) | 20.75  (20.20 – 21.32) | 16.77  (15.62 – 17.99) | 0.90  (0.81 - 1.01) |
| Other | 0.54  (0.48 – 0.61) | 0.55  (0.48 – 0.63) | 0.43  (0.30 – 0.60) | 0.91  (0.62 - 1.34) |

aOR, adjusted odds ratio; aRR, adjusted risk ratio; Hosp, acute care hospitalization; SNF, skill nursing factility; ICF, intermediate care facility.

* adjusted risk ratio and 95% CI reported for length of stay
