# Supplementary material for: In-hospital outcomes and 30-day readmission rates among ischemic and hemorrhagic stroke patients with delirium
Source: PLoS One. 2019 Nov 14;14(11):e0225204. doi: 10.1371/journal.pone.0225204 (PMC6855446; doi:10.1371/journal.pone.0225204)
Supplement: S4 Table — (DOCX) [file pone.0225204.s004.docx]

**S4 Table.** **Rate of readmission and in-hospital outcomes among readmitted stroke patients with and without delirium by stroke-type.**

(a) Ischemic Stroke

|  | **Total**  **(n = 1,823,495)** | **No Delirium**  **(n = 1,711,155)** | **Delirium**  **(n = 112,340)** | **aRR**  **(95% CI)** |
| --- | --- | --- | --- | --- |
| Readmission Rate, % (95% CI) | 12.44  (12.33 – 12.55) | 12.15  (12.04 – 12.26) | 16.76  (16.38 – 17.16) | 1.14  (1.11 - 1.16) |
| Number of readmissions | **Total**  **(n = 251,653)** | **No Delirium**  **(n = 230,489)** | **Delirium**  **(n = 21,164)** | **aOR / aRR**  **(95% CI)*** |
| Died, %(95% CI) | 6.54  (6.36 – 6.73) | 6.26  (6.08 – 6.45) | 9.56  (8.89 – 10.28) | 1.35  (1.23 – 1.47) |
| Length of Stay, mean(SE) | 6.50 (0.03) | 6.42 (0.04) | 7.40 (0.10) | 1.05  (1.03 – 1.08) |
| Discharge Disposition, % (95% CI) | | | | |
| Home incl. Home with Home Health | 49.79  (49.31 – 50.26) | 51.22  (50.72 – 51.72) | 34.12  (32.93 – 35.34) | Reference |
| Transfer (Hosp /SNF/ICF/Other) | 42.98  (42.52 – 43.44) | 41.82  (41.34 – 42.30) | 55.66  (54.40 – 56.91) | 1.63  (1.53 - 1.74) |
| Died | 6.54  (6.36 – 6.73) | 6.26  (6.08,6.45) | 9.56  (8.89 – 10.28) | 1.73  (1.56 - 1.93) |
| Other | 0.69  (0.64 – 0.75) | 0.7  (0.64 – 0.76) | 0.66  (0.51 – 0.85) | 1.31  (0.99 - 1.73) |
| Number of readmissions with delirium | 29,665 | 24,119 | 5,546 |  |
| Proportion of readmissions with delirium, % (95% CI) | 11.79  (11.53 – 12.05) | 10.46  (10.22 – 10.71) | 26.20  (25.16 – 27.27) | 1.96  (1.87 - 2.06) |

aOR, adjusted odds ratio; aRR, adjusted risk ratio; Hosp, acute care hospitalization; SNF, skill nursing factility; ICF, intermediate care facility.

* adjusted risk ratio and 95% CI reported for length of stay

(b) Intracerebral Hemorrhage

|  | **Total**  **(n = 185,921)** | **No Delirium**  **(n = 167,550)** | **Delirium**  **(n = 18,371)** | **aRR**  **(95% CI)** |
| --- | --- | --- | --- | --- |
| Readmission Rate, % (95% CI) | 13.70  (13.40 – 13.99) | 13.32  (13.02 – 13.63) | 17.08  (16.14 – 18.06) | 1.12  (1.06 – 1.19) |
| Number of readmissions | **Total**  **(n = 28,390)** | **No Delirium**  **(n = 24,876)** | **Delirium**  **(n = 3514)** | **aOR / aRR**  **(95% CI)*** |
| Died, %(95% CI) | 7.65  (7.09 – 8.25) | 7.60  (7.01 – 8.24) | 8.00  (6.50 – 9.82) | 1.03  (0.80 – 1.31) |
| Length of Stay, mean(SE) | 7.82 (0.11) | 7.69 (0.11) | 8.78 (0.32) | 1.06  (0.99 – 1.14) |
| Discharge Disposition, % (95% CI) | | | | |
| Home incl. Home with Home Health | 38.98  (37.84 – 40.13) | 40.24  (39.03 – 41.47) | 30.04  (27.32 – 32.90) |  |
| Transfer (Hosp /SNF/ICF/Other) | 52.71  (51.56 – 53.86) | 51.48  (50.27 – 52.70) | 61.40  (58.34 – 64.37) | 1.50  (1.28 - 1.75) |
| Died | 7.65  (7.09 – 8.25) | 7.6  (7.01 – 8.24) | 8.00  (6.50 – 9.82) | 1.26  (0.96 - 1.65) |
| Other | 0.66  (0.52 – 0.83) | 0.67  (0.53 – 0.86) | 0.56  (0.31 – 1.00) | 0.94  (0.48 - 1.87) |
| Number of readmissions with delirium | 4,770 | 3,756 | 1,014 |  |
| Proportion of readmissions with delirium, % (95% CI) | 16.80  (16.05 – 17.58) | 15.10  (14.29 – 15.94) | 28.86  (26.12 – 31.77) | 1.78  (1.58 – 2.01) |

aOR, adjusted odds ratio; aRR, adjusted risk ratio; Hosp, acute care hospitalization; SNF, skill nursing factility; ICF, intermediate care facility.

* adjusted risk ratio and 95% CI reported for length of stay

(c) Subarachnoid Hemorrhage

|  | **Total**  **(n = 77,694)** | **No Delirium**  **(n = 70,771)** | **Delirium**  **(n = 6,923)** | **aRR**  **(95% CI)** |
| --- | --- | --- | --- | --- |
| Readmission Rate, % (95% CI) | 11.48  (11.01 – 11.96) | 11.20  (10.71 – 11.72) | 14.27  (12.74 – 15.94) | 1.08  (0.96 – 1.22) |
| Number of readmissions | **Total**  **(n = 9,787)** | **No Delirium**  **(n = 8,697)** | **Delirium**  **(n = 1,089)** | **aOR / aRR**  **(95% CI)*** |
| Died, %(95% CI) | 4.64  (3.92 – 5.48) | 4.31  (3.57 – 5.18) | 7.28  (4.90 – 10.67) | 1.64  (0.96 – 2.79) |
| Length of Stay, mean(SE) | 7.50 (0.19) | 7.30 (0.19) | 9.09 (0.62) | 1.13  (1.00 – 1.27) |
| Discharge Disposition, % (95% CI) | | | | |
| Home incl. Home with Home Health | 58.6  (56.55 – 60.62) | 60.97  (58.74 – 63.15) | 39.66  (34.29 – 45.28) | Reference |
| Transfer (Hosp /SNF/ICF/Other) | 36.49  (34.44 – 38.58) | 34.49  (32.31 – 36.73) | 52.43  (46.85 – 57.94) | 2.16  (1.64 - 2.84) |
| Died | 4.64  (3.92 – 5.48) | 4.31  (3.57 – 5.18) | 7.28  (4.90 – 10.67) | 3.12  (1.77 - 5.53) |
| Other | 0.28  (0.17 – 0.47) | 0.24  (0.14 – 0.41) | 0.64  (0.18 – 2.22) | 2.64  (0.87 - 7.99) |
| Number of readmissions with delirium | 1,230 | 985 | 245 |  |
| Proportion of readmissions with delirium, % (95% CI) | 12.57  (11.32 – 13.93) | 11.33  (10.08 – 12.71) | 22.47  (18.59 – 26.88) | 1.70  (1.36 – 2.11) |

aOR, adjusted odds ratio; aRR, adjusted risk ratio; Hosp, acute care hospitalization; SNF, skill nursing factility; ICF, intermediate care facility.

* adjusted risk ratio and 95% CI reported for length of stay
