# Supplementary material for: In-hospital outcomes and 30-day readmission rates among ischemic and hemorrhagic stroke patients with delirium
Source: PLoS One. 2019 Nov 14;14(11):e0225204. doi: 10.1371/journal.pone.0225204 (PMC6855446; doi:10.1371/journal.pone.0225204)
Supplement: S5 Table — Results of sensitivity analyses for association of concurrent mild cognitive impairment or dementia (MCID) with (a) delirium diagnosis and (b) in-hospital and 30-day outcomes among stroke discharges. (DOCX) [file pone.0225204.s005.docx]

**S5 Table. Results of sensitivity analyses for association of concurrent mild cognitive impairment or dementia (MCID) with (a) delirium diagnosis and (b) in-hospital and 30-day outcomes among stroke discharges.** Concurrent MCI / dementia diagnosis was identified using validated ICD-9 diagnosis codes (dementia ICD-9 codes: 290.11 – 290.13, 290.2, 290.3, 290.41 – 290.43, 290.8, 290.9, 291, 292, 292.11, 292.12, 292.2, 292.81, 292.82, 293, 293.1, 293.81 – 293.84, 293.89, 293.9, 348.3, 348.31, 348.39, 349.82, 780.02, 780.09, 780.97; MCI ICD-9 codes: 331.83, 780.93) [1].

(a)

|  | **Total**  **(n=3,107,437)** | **No Delirium**  **(n=2,875,938)** | **Delirium**  **(n=231,500)** | **OR**  **(95% CI)** |
| --- | --- | --- | --- | --- |
| MCI / Dementia | 6.33 | 5.71 | 14.07 | 2.70 (2.64–2.78) |

(b)

|  | **aOR / aRR**  **(95% CI)**  **Without adjustment with concurrent MCI / dementia diagnosis** | **aOR / aRR**  **(95% CI)**  **With adjustment of concurrent MCI / dementia diagnosis** |
| --- | --- | --- |
| **In-Hospital Outcomes** |  |  |
| Died, %(95% CI) | 1.36 (1.31 – 1.41)* | 1.38 (1.33 – 1.43)* |
| Length of Stay, mean(SE) | 1.24 (1.23 – 1.25)^†^ | 1.24 (1.23 – 1.25)^†^ |
| Discharge Disposition, % (95% CI) | | |
| Home incl. Home with Home Health | Reference | Reference |
| Transfer (Hosp/SNF/ICF/Other) | 1.79 (1.75 – 1.83)* | 1.74 (1.71 – 1.78)* |
| Died | 1.88 (1.81 – 1.95)* | 1.88 (1.81 – 1.95)* |
| Other | 1.53 (1.41 – 1.66)* | 1.53 (1.41 – 1.66)* |
| **30-Day Outcomes** |  |  |
| Readmission Rate, % (95% CI) | 1.13 (1.11 – 1.15)^††^ | 1.13 (1.11 – 1.16)^††^ |
| Died, %(95% CI) | 1.32 (1.21 – 1.44)^*^ | 1.33 (1.22 – 1.45)* |
| Length of Stay, mean(SE) | 1.08 (1.05 – 1.11)^†^ | 1.08 (1.04 – 1.10)^†^ |
| Discharge Disposition, % (95% CI) | | |
| Home incl. Home with Home Health | Reference | Reference |
| Transfer (Hosp/SNF/ICF/Other) | 1.68 (1.58 – 1.79)* | 1.66 (1.56 – 1.76)* |
| Died | 1.72 (1.56 – 1.90)* | 1.73 (1.56 – 1.91)* |
| Other | 1.29 (1.00 – 1.67)* | 1.28 (0.99 – 1.65)* |
| Delirium on readmission | 1.94 (1.86 – 2.02)^††^ | 1.91 (1.83 – 1.99)^††^ |

All models controlled for: (1) *Demographic Variables*: Age, Sex, Insurance Type, Income Quartile by Zip Code of Residence (2) *Comorbidities*: Charlson Co-morbidity Index, Number of Chronic Diseases, Atrial Fibrillation, Coagulopathies, Hypertension, Peripheral Vascular Disease, Valvular Disease, Diseases of Pulmonary Circulation, Other Neurological Diseases, Depression, Psychiatric Illness, Chronic Lung Disease, Liver Disease, Diabetes Mellitus (with complications), Renal Disease, Electrolyte Imbalance, Anemia, Chronic Blood Loss, Ulcer, Tumor, Obesity, Drug Abuse, Alcohol Abuse (3) *Stroke Type and Treatment Intensity*: Hemicraniectomy / Craniotomy, Extra Ventricular Drain Placement, Gastric Tube, Tracheostomy, Ventilator Support, Intravenous IV tPA, Intra-Arterial Therapy, Stroke Type

* Estimates obtained from multivariable *logistic regression models (Odds Ratio)*

^†^ Estimates obtained from *negative binomial regression models (Risk Ratio)*

^††^ Estimates obtained from *Poisson regression models (Risk Ratio)*

**S5 Table References**

1. St Germaine-Smith C, Metcalfe A, Pringsheim T, Roberts JI, Beck CA, et al. (2012) Recommendations for optimal ICD codes to study neurologic conditions: a systematic review. Neurology 79: 1049-1055.
