# Supplementary material for: In-hospital outcomes and 30-day readmission rates among ischemic and hemorrhagic stroke patients with delirium
Source: PLoS One. 2019 Nov 14;14(11):e0225204. doi: 10.1371/journal.pone.0225204 (PMC6855446; doi:10.1371/journal.pone.0225204)
Supplement: Attachment — Submitted filename: RebuttalLetter.docx [file pone.0225204.s006.docx]

**In-Hospital Outcomes and 30-day Readmission Rates among Ischemic and Hemorrhagic Stroke Patients with Delirium**

Rebuttal Letter to: PONE-D-19-25809, PLOS ONE

**Corresponding Author:**

Farhaan S. Vahidy, PhD MBBS MPH

[Farhaan.Vahidy@uth.tmc.edu](mailto:Farhaan.Vahidy@uth.tmc.edu)

Dear Editor,

We appreciate the opportunity to revise and resubmit our manuscript. We also thank the editorial staff and the reviewers for their time in reviewing our work. We have made significant changes to the manuscript based on their comments and believe that our work has been strengthened by these changes. We hereby provide a detailed response to each point raised by the academic editor and reviewers. In addition to this rebuttal letter labeled as 'Response to Reviewers' in the submission system, we have provided a marked-up copy of our manuscript, highlighting changes made to the original version, labeled as ‘Revised Manuscript with Track Changes' and an unmarked version of our revised manuscript without tracked changes labeled as 'Manuscript'. We look forward to a reassessment of our revised manuscript.

Regards,

Farhaan Vahidy.

**Editorial Comments:**

**Journal Requirements:**

When submitting your revision, we need you to address these additional requirements.

1. Please ensure that your manuscript meets PLOS ONE's style requirements, including those for file naming. The PLOS ONE style templates can be found at <http://www.journals.plos.org/plosone/s/file?id=wjVg/PLOSOne_formatting_sample_main_body.pdf> and <http://www.journals.plos.org/plosone/s/file?id=ba62/PLOSOne_formatting_sample_title_authors_affiliations.pdf>

**Response:** We thank the opportunity to review and update the manuscript to make sure the manuscript meets PLOS ONE's style requirements with appropriate file naming. We have updated manuscript file to meet PLOS ONE’s style requirements, including those for file naming.

2. We note that you have indicated that data from this study are available upon request. PLOS only allows data to be available upon request if there are legal or ethical restrictions on sharing data publicly. For information on unacceptable data access restrictions, please see http://journals.plos.org/plosone/s/data-availability#loc-unacceptable-data-access-restrictions.

In your revised cover letter, please address the following prompts:

a) If there are ethical or legal restrictions on sharing a de-identified data set, please explain them in detail (e.g., data contain potentially identifying or sensitive patient information) and who has imposed them (e.g., an ethics committee). Please also provide contact information for a data access committee, ethics committee, or other institutional body to which data requests may be sent.

b) If there are no restrictions, please upload the minimal anonymized data set necessary to replicate your study findings as either Supporting Information files or to a stable, public repository and provide us with the relevant URLs, DOIs, or accession numbers. Please see http://www.bmj.com/content/340/bmj.c181.long for guidelines on how to de-identify and prepare clinical data for publication. For a list of acceptable repositories, please see http://journals.plos.org/plosone/s/data-availability#loc-recommended-repositories.

We will update your Data Availability statement on your behalf to reflect the information you provide.

**Response:** We thank for the opportunity to update the Data Availability statement. We have removed data availability related text from the manuscript method section [page 3, lines 49-53 in the ‘Revised Manuscript with Track Changes' file] and like to update the Data Availability Statement in the submission system as follow:

Data analyzed in the study are publicly available for purchase from the Central Distributor of the Healthcare Cost Utilization Project (HCUP) of the US Agency for Healthcare Research and Quality for researchers with online Data Use Agreements and completion of the HCUP Data Use Agreement Training (URL: <https://www.distributor.hcup-us.ahrq.gov>; email: HCUPDistributor@AHRQ.gov). The authors used the Nationwide Readmission Database (NRD) of the HCUP for the study for years 2010-2015. Data were obtained through following steps: completion of the web-based HCUP Data Use Agreement (DUA) Training, registration for an account with the online HCUP Central Distributor, completion of the profile using the DUA Training certification code and completion date, submission of an application for purchase of the HCUP NRD data with online acknowledgment of applicable HCUP DUAs, and submission of the payments. Data were delivered to the authors via secure digital download. Detailed information regarding each step is available at <https://www.distributor.hcup-us.ahrq.gov>. Description of data elements for the HCUP NRD is available at <https://www.hcup-us.ahrq.gov/db/nation/nrd/nrddde.jsp>. None of the authors of the present study are affiliated with the HCUP or collected any of the data on behalf of the HCUP or have special access privileges in accessing the HCUP. Data were purchased publicly from the HCUP. All interested researchers can access the data through HCUP in a similar manner through completing and submitting an application for purchasing HCUP Databases at the HCUP Central Distributor (https://www.distributor.hcup-us.ahrq.gov).

3. Thank you for stating the following in the Acknowledgments Section of your manuscript:

'We acknowledge the infrastructural support provided by the Biostatistics/ Epidemiology/Research Design (BERD) component of the Center for Clinical and Translational Sciences (CCTS) for this project. CCTS is mainly funded by a grant (UL1 TR000371) from the National Center for Advancing Translational Sciences (NCATS), awarded to University of Texas Health Science Center at Houston.'

We note that you have provided funding information that is not currently declared in your Funding Statement. However, funding information should not appear in the Acknowledgments section or other areas of your manuscript. We will only publish funding information present in the Funding Statement section of the online submission form.

Please remove any funding-related text from the manuscript and let us know how you would like to update your Funding Statement. Currently, your Funding Statement reads as follows:

'The author(s) received no specific funding for this work.'

**Response:** We thank for the opportunity to update the Acknowledgments Section. We have removed the funding-related text from the manuscript [page 16, lines 307-313 in the 'Revised Manuscript with Track Changes']. We like to update our Funding Statement as follow:

We acknowledge the infrastructural support provided by the Biostatistics/ Epidemiology/Research Design (BERD) component of the Center for Clinical and Translational Sciences (CCTS) for this project. CCTS is mainly funded by a grant (UL1 TR000371) from the National Center for Advancing Translational Sciences (NCATS), awarded to University of Texas Health Science Center at Houston.

**Reviewers' Responses to Questions and Comments to the Author:**

1. Is the manuscript technically sound, and do the data support the conclusions?

The manuscript must describe a technically sound piece of scientific research with data that supports the conclusions. Experiments must have been conducted rigorously, with appropriate controls, replication, and sample sizes. The conclusions must be drawn appropriately based on the data presented.

Reviewer #1: Yes

Reviewer #2: Partly

**Response:** Thank you for reviewing our work.

2. Has the statistical analysis been performed appropriately and rigorously?

Reviewer #1: Yes

Reviewer #2: Yes

**Response:** Thank you for reviewing our work.

3. Have the authors made all data underlying the findings in their manuscript fully available?

The PLOS Data policy requires authors to make all data underlying the findings described in their manuscript fully available without restriction, with rare exception (please refer to the Data Availability Statement in the manuscript PDF file). The data should be provided as part of the manuscript or its supporting information, or deposited to a public repository. For example, in addition to summary statistics, the data points behind means, medians and variance measures should be available. If there are restrictions on publicly sharing data—e.g. participant privacy or use of data from a third party—those must be specified.

Reviewer #1: Yes

Reviewer #2: Yes

**Response:** Thank you for reviewing our work.

4. Is the manuscript presented in an intelligible fashion and written in standard English?

PLOS ONE does not copyedit accepted manuscripts, so the language in submitted articles must be clear, correct, and unambiguous. Any typographical or grammatical errors should be corrected at revision, so please note any specific errors here.

Reviewer #1: Yes

Reviewer #2: Yes

**Response:** Thank you for reviewing our work.

5. Review Comments to the Author

Reviewer #1: Authors have performed a comprehensive study using Nationwide Claims data, assessing the prevalence of delirium and its association with outcomes among IS and hemorrhagic stroke patients. I have following comments:

**Response:** Thank you for reviewing our work.

1. Why did the authors select all-cause readmissions, instead of stroke related readmissions? I would suggest performing additional association of readmission 2/2 recurrent stroke or worsening of stroke symptoms with defined outcomes.

**Response:** We thank the opportunity to explain this further. We selected all-cause readmissions because validated methodologies for identification of readmissions due to recurrent strokes or worsening of stroke symptoms using administrative database have not been developed. A considerable degree of variability exists in how hospital events are coded across the US, and a primary diagnosis of ‘stroke’ on readmission does not necessarily identify a recurrent stroke. Similarly, administrative data do not allow for validly identifying readmissions caused by worsening of stroke symptoms.

However, based on reviewer’s suggestion, we conducted additional analyses for 30-day readmission rate among patients with same primary discharge diagnosis as that of the index admission. Overall, we found that 18.9% readmissions had the same primary diagnosis as that of the index admission. This proportion was higher among non-delirious patients as compared to delirious patients (19.5% vs. 12.5%). The rate of readmission for same primary diagnoses among non-delirious patients was 2.4% where as it was 2.1% among delirious patients. Though the interpretational value of these differences is limited. These findings may suggest that delirious patients may have a higher likelihood of non-stroke related readmissions.

2. For patients with ICH, did the authors have specific data to differentiate the associations specifically for hemorrhagic conversion and parenchymal hematomas?

**Response:** Thank you for this comment. Administrative data do not allow for direct identification of hemorrhagic conversions. However, we conducted additional analyses for concurrent diagnosis of acute ischemic stroke with or without thrombolytic therapies (tPA or intraarterial therapy) among patients with a primary ICH diagnosis and found that proportion of patients with concurrent ischemic stroke diagnosis was significantly higher in delirious ICH patients as compared to non-delirious ICH patients (4.95% vs. 3.38%). Similarly, proportion of patients with concurrent diagnoses of both ischemic stroke and thrombolytic therapy was higher among delirious patients as compared to non-delirious patients (0.1% vs. 0.07%). These differences should be interpreted with caution. Though these may seem to suggest that patients with hemorrhagic conversions have higher likelihood of developing delirium, it is also likely that administrative coding for ischemic stroke may represent a prior ischemic episode.

Reviewer #2: The present study assesses the consequences of in-hospital delirium in patients with stroke. The strengths of the study include the large patient population and the comprehensive statistical analysis. Major limitations are the utilisation of icd codes for the detection of diseases, comorbidities, and complications, the retrospective nature and the lack of data on the cognitive status of patients. Also, there was no adjustment made for possible comorbid dementia or mild cognitive impairment which are maybe the most important risk factors of delirium during hospitalisation (adjustment was made for ‘other’ neurological diseases in general). There is also no adjustment for chronic medications, for instance benzodiazepine use increases risk of delirium, too. All these very important limitations derive from the retrospective nature of the study based on diagnostic codes and are almost impossible to overcome. Consequently, the results of the study may be used primarily for hypotheses generation. All these have to be emphasized by the authors, as well as the need for further prospective studies with bedside assessment protocols.

**Response:** We thank the reviewer for their time and effort and pointing out the strengths and major limitations. We have added text in the discussion section to reflect these major limitations. Please see pages 15 (lines 298-291, 296-297 in the 'Revised Manuscript with Track Changes'). We used the published algorithm for administrative claims databases to identify delirium cases [Reference 1]. This algorithm was validated against the confusion assessment method for delirium assessment and includes ICD-9 diagnosis codes which are overlapping with validated ICD-9 diagnosis codes for diagnosis of dementia [Reference 2] (overlapping ICD-9 diagnosis codes: 290.11, 290.12, 290.13, 290.2, 290.3, 290.41, 290.42, 290.43, 290.8, and 290.9).

We have conducted additional analyses to examine association of mild cognitive impairment or dementia (MCID) with delirium diagnosis and results are shown in the table below. As expected, stroke patients with delirium had higher odds of having MCID diagnosis compared to those without delirium.

|  | **Total**  **(n=3,107,437)** | **No Delirium**  **(n=2,875,938)** | **Delirium**  **(n=231,500)** | **OR**  **(95% CI)** |
| --- | --- | --- | --- | --- |
| MCI / Dementia | 6.33 | 5.71 | 14.07 | 2.70 (2.64–2.78) |

We also conducted additional analyses for in-hospital and 30-day outcomes with addition of MCID diagnosis in adjusted models and results are shown in the table below. We found that adjusted odds ratios and risk ratios for in-hospital and 30-day outcomes in models with additional MCID diagnosis comparing delirium versus non-delirium were similar to those models without additional MCID diagnosis.

|  | **aOR / aRR**  **(95% CI)**  **Without adjustment with concurrent dementia diagnosis** | **aOR / aRR**  **(95% CI)**  **With adjustment of concurrent dementia diagnosis** |
| --- | --- | --- |
| **In-Hospital Outcomes** |  |  |
| Died, %(95% CI) | 1.36 (1.31 – 1.41)* | 1.38 (1.33 – 1.43)* |
| Length of Stay, mean(SE) | 1.24 (1.23 – 1.25)^†^ | 1.24 (1.23 – 1.25)^†^ |
| Discharge Disposition, % (95% CI) | | |
| Home incl. Home with Home Health | Reference | Reference |
| Transfer (Hosp/SNF/ICF/Other) | 1.79 (1.75 – 1.83)* | 1.74 (1.71 – 1.78)* |
| Died | 1.88 (1.81 – 1.95)* | 1.88 (1.81 – 1.95)* |
| Other | 1.53 (1.41 – 1.66)* | 1.53 (1.41 – 1.66)* |
| **30-Day Outcomes** |  |  |
| Readmission Rate, % (95% CI) | 1.13 (1.11 – 1.15)^††^ | 1.13 (1.11 – 1.16)^††^ |
| Died, %(95% CI) | 1.32 (1.21 – 1.44)^*^ | 1.33 (1.22 – 1.45)* |
| Length of Stay, mean(SE) | 1.08 (1.05 – 1.11)^†^ | 1.08 (1.04 – 1.10)^†^ |
| Discharge Disposition, % (95% CI) | | |
| Home incl. Home with Home Health | Reference | Reference |
| Transfer (Hosp/SNF/ICF/Other) | 1.68 (1.58 – 1.79)* | 1.66 (1.56 – 1.76)* |
| Died | 1.72 (1.56 – 1.90)* | 1.73 (1.56 – 1.91)* |
| Other | 1.29 (1.00 – 1.67)* | 1.28 (0.99 – 1.65)* |
| Delirium on readmission | 1.94 (1.86 – 2.02)^††^ | 1.91 (1.83 – 1.99)^††^ |

All models controlled for: (1) *Demographic Variables*: Age, Sex, Insurance Type, Income Quartile by Zip Code of Residence (2) *Comorbidities*: Charlson Co-morbidity Index, Number of Chronic Diseases, Atrial Fibrillation, Coagulopathies, Hypertension, Peripheral Vascular Disease, Valvular Disease, Diseases of Pulmonary Circulation, Other Neurological Diseases, Depression, Psychiatric Illness, Chronic Lung Disease, Liver Disease, Diabetes Mellitus (with complications), Renal Disease, Electrolyte Imbalance, Anemia, Chronic Blood Loss, Ulcer, Tumor, Obesity, Drug Abuse, Alcohol Abuse (3) *Stroke Type and Treatment Intensity*: Hemicraniectomy / Craniotomy, Extra Ventricular Drain Placement, Gastric Tube, Tracheostomy, Ventilator Support, Intravenous IV tPA, Intra-Arterial Therapy, Stroke Type

* Estimates obtained from multivariable *logistic regression models (Odds Ratio)*

^†^ Estimates obtained from *negative binomial regression models (Risk Ratio)*

^††^ Estimates obtained from *Poisson regression models (Risk Ratio)*

Reference 1: Kim DH, Lee J, Kim CA, Huybrechts KF, Bateman BT, et al. (2017) Evaluation of algorithms to identify delirium in administrative claims and drug utilization database. Pharmacoepidemiol Drug Saf 26: 945-953.

Reference 2: St Germaine-Smith C, Metcalfe A, Pringsheim T, Roberts JI, Beck CA, et al. (2012) Recommendations for optimal ICD codes to study neurologic conditions: a systematic review. Neurology 79: 1049-1055.

6. PLOS authors have the option to publish the peer review history of their article (what does this mean?). If published, this will include your full peer review and any attached files.

If you choose “no”, your identity will remain anonymous but your review may still be made public.

Do you want your identity to be public for this peer review? For information about this choice, including consent withdrawal, please see our Privacy Policy.

Reviewer #1: Yes: Konark Malhotra

Reviewer #2: No

**Response:** Thank you for reviewing our work.

If reviewer comments were submitted as an attachment file, they will be attached to this email and accessible via the submission site. Please log into your account, locate the manuscript record, and check for the action link "View Attachments". If this link does not appear, there are no attachment files to be viewed.

**Response:** Thank you for reviewing our work.

**Additional Requirements:**

While revising your submission, please upload your figure files to the Preflight Analysis and Conversion Engine (PACE) digital diagnostic tool, https://pacev2.apexcovantage.com/. PACE helps ensure that figures meet PLOS requirements. To use PACE, you must first register as a user. Registration is free. Then, login and navigate to the UPLOAD tab, where you will find detailed instructions on how to use the tool. If you encounter any issues or have any questions when using PACE, please email us at figures@plos.org. Please note that Supporting Information files do not need this step.

**Response:** Thank you for reviewing our work. We have used the PACE digital diagnostic tool and converted our figure files as per instructions in the tool and submitted those figure files in the PLOS ONE submission system.
