# Supplementary material for: In-hospital outcomes and 30-day readmission rates among ischemic and hemorrhagic stroke patients with delirium
Source: PLoS One. 2019 Nov 14;14(11):e0225204. doi: 10.1371/journal.pone.0225204 (PMC6855446; doi:10.1371/journal.pone.0225204)
Supplement: Attachment — Submitted filename: RebuttalLetter.docx [file pone.0225204.s007.docx]

**In-Hospital Outcomes and 30-day Readmission Rates among Ischemic and Hemorrhagic Stroke Patients with Delirium**

Rebuttal Letter to: PONE-D-19-25809R1, PLOS ONE

**Corresponding Author:**

Farhaan S. Vahidy, PhD MBBS MPH

[Farhaan.Vahidy@uth.tmc.edu](mailto:Farhaan.Vahidy@uth.tmc.edu)

Dear Editor,

We appreciate the opportunity to revise and resubmit our manuscript revision #1. We have made changes to the manuscript as per the reviewers’ comments and responses are provided below. In addition to this rebuttal letter labeled as 'Response to Reviewers' in the submission system, we have provided a marked-up copy of our manuscript, highlighting changes made to the original version, labeled as ‘Revised Manuscript with Track Changes' and an unmarked version of our revised manuscript without tracked changes labeled as 'Manuscript'. We look forward to a quick reassessment of our revised manuscript.

Regards,

Farhaan Vahidy.

**Reviewers' Responses to Questions and Comments to the Author:**

Reviewer #1: All comments have been addressed by the authors.

No further modifications in the manuscript are needed.

**Response:** We thank the reviewer for their time and effort.

Reviewer #2: Thank you for addressing the comments with such a comprehensive fashion. Please include the additional analysis conserving MCI and dementia (association and outcomes) as supporting information.

**Response:** We thank the reviewer for their time and effort. We have added the additional analysis that compares outcomes with and without MCID as a covariate (S5 Table). Corresponding revisions have been made to the manuscript as well. Please see pages 15 (lines 285-289, lines 377-379).
